# Analysis in the influence factors of urethroplasty in DSD

**DOI:** 10.1186/s12894-022-01080-x

**Published:** 2022-08-10

**Authors:** Jing Yu, Ning Sun, Hongcheng Song, Minglei Li, Lele Li, Chunxiu Gong, Weiping Zhang

**Affiliations:** Department of Urology, National Center for Children’s Health, Beijing Children’s Hospital Affiliated to Capital Medical University, Beijing, 100045 China

**Keywords:** Analysis, Influencing factors, Urethroplasty, Disorders of sex development

## Abstract

**Background:**

At present, there is no specific research on the factors affecting the success rate of urethroplasty in patients with DSD. The purpose of this study is to explore the factors affecting the success of urethroplasty in DSD patients, and to provide some reference for the surgical treatment of DSD patients undergoing urethroplasty.

**Method:**

We reviewed patients with DSD who underwent urethroplasty from January 2016 to December 2019 retrospectively. Patients were divided into four groups: the successful group, the urethrocutaneous fistula group, the urethral diverticulum group, and the urethral stricture group. Risk factors were determined from the following data included the DSD classification, the age of first operation, length of urethral defect, degree of hypospadias, cryptorchidism, micropenis, gonad type, hormone therapy before operation, transposition of penis and scrotum, surgical strategy, urethral covering material, and postoperative catheter removal time. We explored the difference of each factor between four groups through the comparative study of single factor and multifactor logistic regression analysis of related factors.

**Result:**

122 cases were enrolled in this group (n = 122), 12 cases were lost to follow-up. Median follow-up was 28 months (12–55 months).We found the success rate of operation decreased with longer urethral defect (B = − 0.473, P = 0.005). The success rate of operation was higher in staged operation and TPIT (TPIT = Transverse Preputial Island Tube operation)-related operation than primary operation (B = 1.238, P = 0.006) and TPIT-nonrelated operation (B = 2.293, P = 0.001). Although there was a significant difference between the age of the first operation and the occurrence of urethrocutaneous fistula (P = 0.006 < 0.05), there was no significant difference in logistic regression analysis (P = 0.161 > 0.05). The incidence of urethrocutaneous fistula was lower in TPIT-related operation than in TPIT-nonrelated operation (B = − 2.507, P = 0.000). The incidence of postoperative urethral diverticulum was lower in staged operation than in primary operation (B = − 1.737, P = 0.015).

**Conclusion:**

For patients with disorder of sex development undergoing urethroplasty, the length of urethral defect is an independent risk factor affecting both the success rate of operation and the urethrocutaneous fistula. The age of the first operation has a statistically significant effect on the occurrence of postoperative urethrocutaneous fistula, but it is not an independent factor. Urethrocutaneous fistula is less found in TPIT-related operation in the study. Staged operation is an independent protective factor for postoperative urethral diverticulum compared with one-stage operation but isn’t related to urethrocutaneous fistula.

## Background

Disorders of sex development (DSD) is a group of diseases caused by congenital chromosomal, gonadal or sexual anatomical structural abnormalities. The disease spectrum of DSD is extensive, and the pathophysiological changes and clinical manifestations are various. At present, the incidence of DSD is 1/4000 [1], which is usually found in newborn or puberty [2]. Because it involves gender assignment, the treatment of DSD requires the teamwork of pediatric endocrinologist, pediatric psychologist, and pediatric surgeon. Moreover, the gonads and genitals show different manifestations in DSD cases, which makes the repair of genitals more complex, so there is no standard surgical strategy [3]. At present, there is no specific research on the risk factors affecting the success rate of urethroplasty in patients with DSD. Because most of the DSD patients which were performed urethroplasty are complicated with severe hypospadias, most people think that the risk factors of urethroplasty should be the same as those of severe hypospadias. However, hypospadias in DSD patients tends to be more severe, and are usually complicated with mirospenis, bifid scrotum, undescended testis. Thus, there is no standard surgical strategy and each case tends to be treated individually. The purpose of this study is to explore the risk factors affecting the success of urethroplasty in DSD patients, and to provide some references for the surgical treatment for DSD patients.

## Patients and methods

### General data

After the experimental protocol was approved by the Ethics Committee of Beijing Children’s Hospital, Capital Medical University (No. 2018-209). We reviewed 122 patients with DSD who were performed urethroplasty from January 2016 to December 2019 retrospectively. We investigated the DSD classification, the age of first operation, length of urethral defect, degree of hypospadias, cryptorchidism, micropenis, gonad type, hormone therapy before operation, transposition of penis and scrotum, surgical strategy, urethral covering material and postoperative catheter removal time. The outcome was determined by outpatient re-examination and telephone follow-up to learn about post-urethroplasty complications. 12 cases were lost to follow-up and 110 cases completed follow-up. The median follow-up was 28 months with the shortest being 12 months and the longest being 55 months.

#### Inclusion criteria

A. DSD were diagnosed based on the diagnostic criteria of European Pediatric Endocrine Association (ESPE) and Lawson Wilkins Pediatric Endocrine Association (LWPES).
B. According to the results of gonad, chromosome, SRY gene, HCG stimulation test, HMG stimulation test, and imaging examination, gender assignment was decided by endocrinology, urology, psychological experts. Patients had the same conditions were conducted urethroplasty and raised as male.
C. The participant and their parents in the study agreed to participate in the study and signed a ethical informed consent form.

#### Exclusion criteria

Clinical data (clinical manifestations, chromosome reports, auxiliary examination results, surgical records) are not incomplete.

### Main surgical methods

Surgical methods were decided according to the condition of the prepuce and the defect of the urethra. One-stage operation method was selected if the length of prepuce can meet the needs of the defect of the urethra. K was used in the patients with the dysplastic prepuce. However, Byars staged operation was performed in the patients with the dysplastic prepuce when staged operation was selected.

#### One-stage operation

**Fig. 1:**
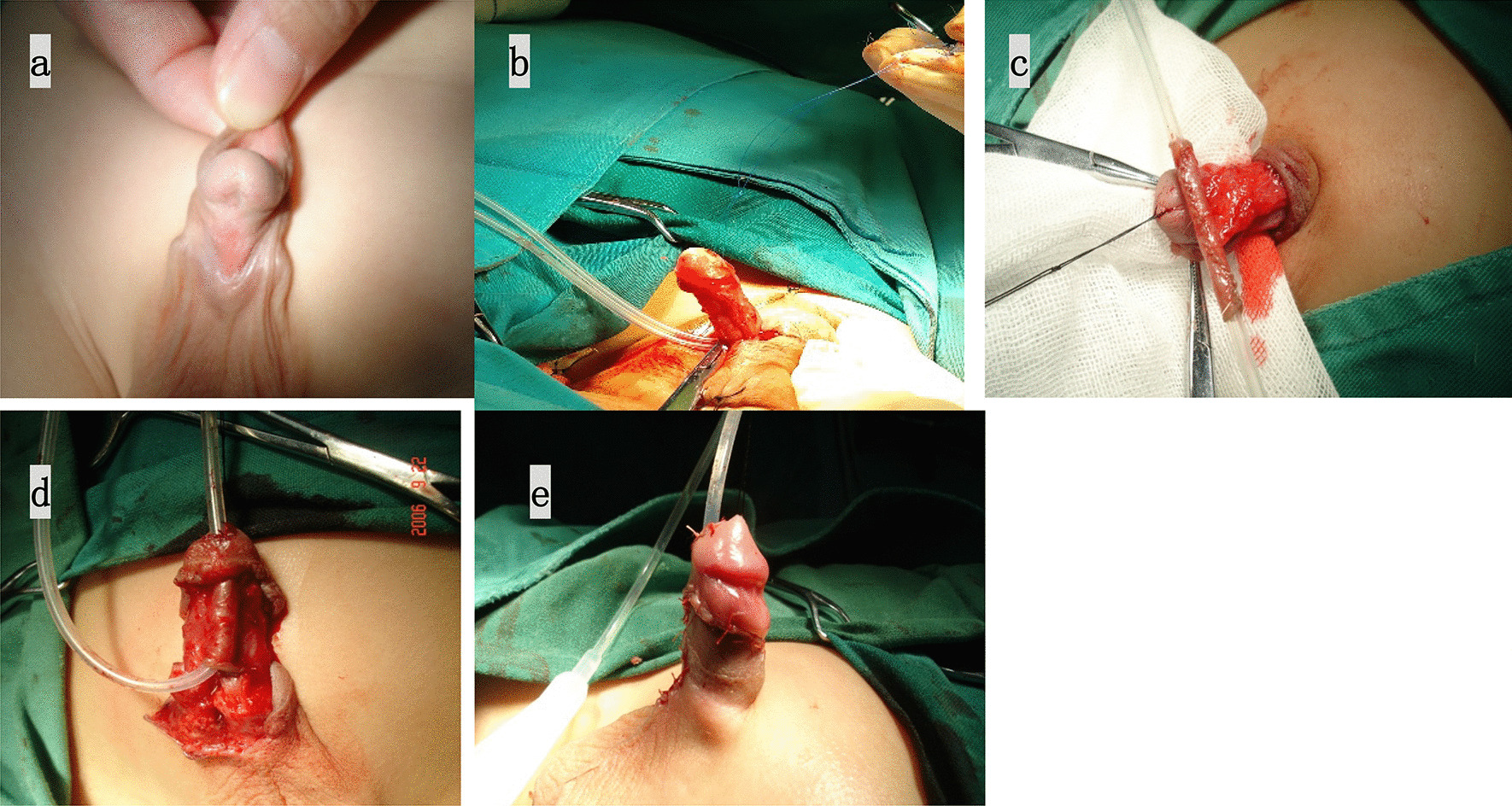
TPIT operation **a** Preoperation appearance; **b** Dorsal plication was performed to completely correct severe chordee; **c** rectangular flap was harvested from the inner prepuce and the mobilized foreskin was rolled into a tube over a catheter. **d** The neourethra was anastomosed with the native urethra and the distal meatus was attached to the top of the glans with interrupted fine sutures; **e** The neourethra was covered by flap

**Fig. 2:**
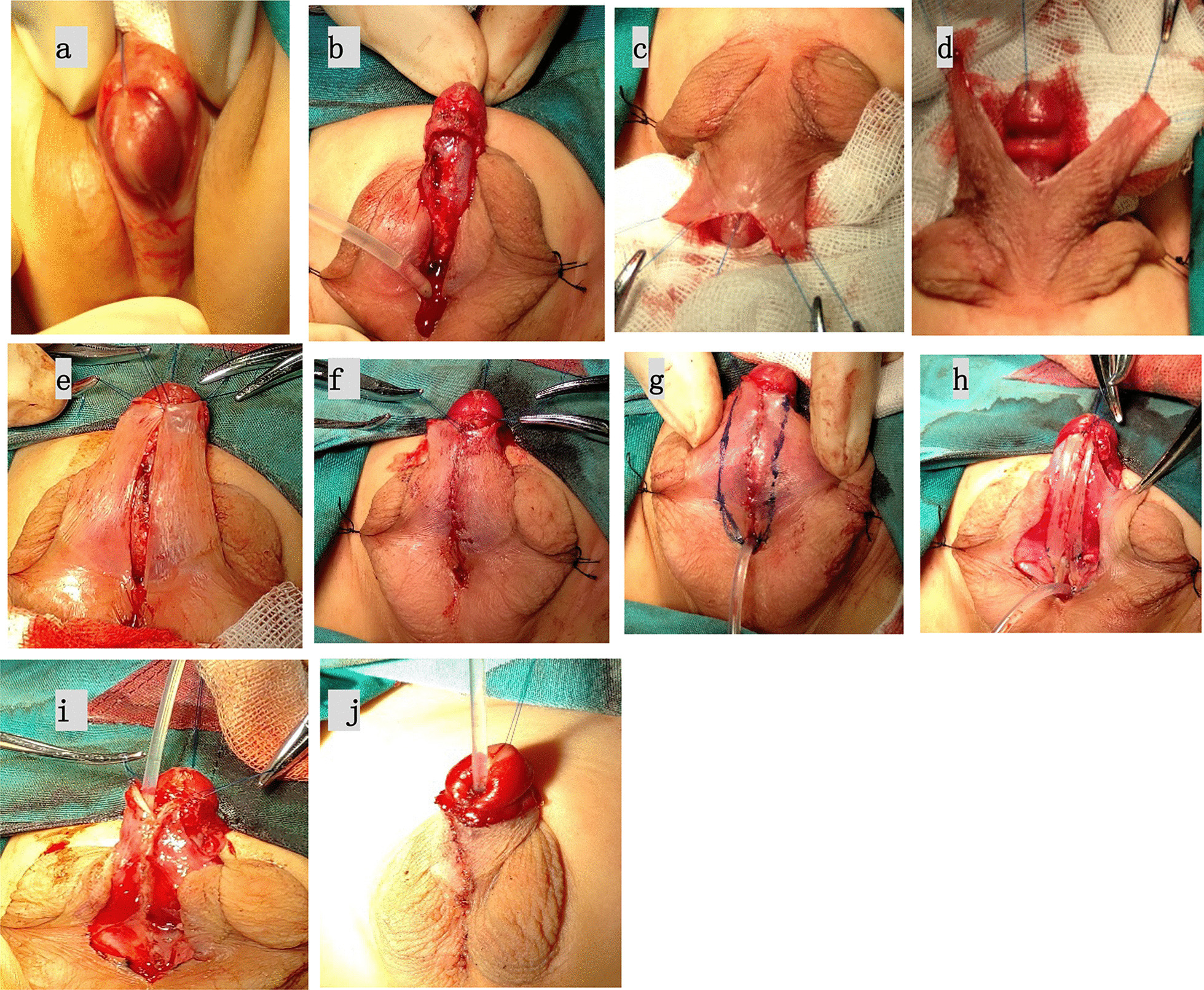
Koyanagi operation **a** preoperative appearance; **b** Penile degloving; **c**, **d** The dorsal median longitudinal cut of the foreskin is indicated and transferred ventrally;**e**–**i** Urethroplasty, first by closing the medial **e**, **f** and then the lateral edges of the wing flaps over the inlying urethral catheter (**g**, **h**, **i**); **j** the neourethra was covered by flap

- *Transverse preputial island tube operation (TPIT)* The TPIT procedure was executed as previously described in the literature [4]. The procedure can be seen in Fig. 1.
- *Koyanagi operation* The procedure was conducted as previously described in the literature [5]. The procedure can be seen in Fig. 2.

#### Staging operation

**Fig. 3:**
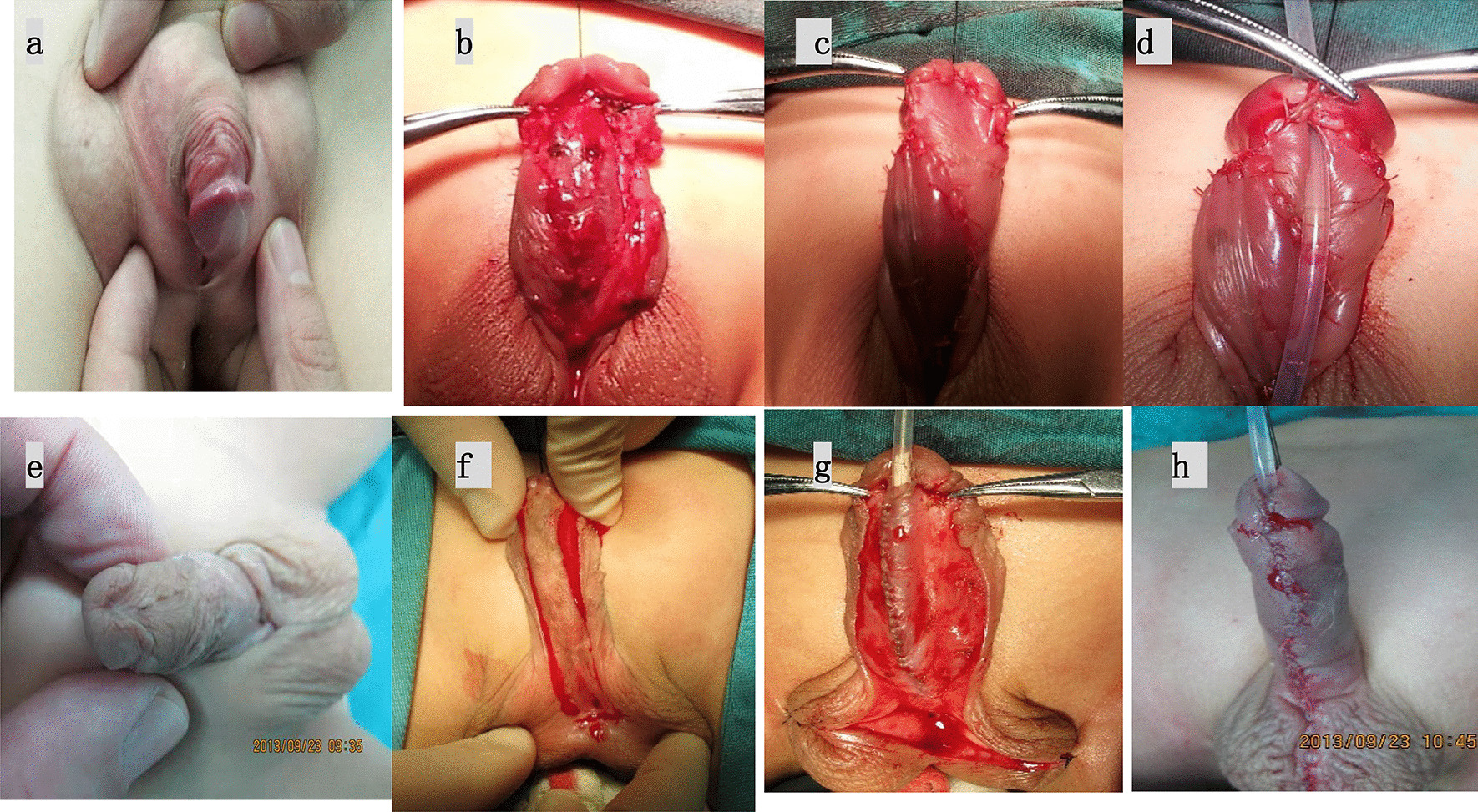
Byars staging operation **a** preoperative appearance; **b** Degloving and chordee release; **c**, **d** Preputial skin flap (Byar’s flap) sutured to cover the raw area created on retraction of the “glans wings” and on areas created after release of chordee. **e** Preoperative appearance of 2nd staged operation; **f** Parallel lines were marked on the ventral side of the penis according to the catheter circumference and urethra defect. **g** The rotated skin was tubularized around the catheter to make the new urethra. **h** The neourethra was covered by flap

**Fig. 4:**
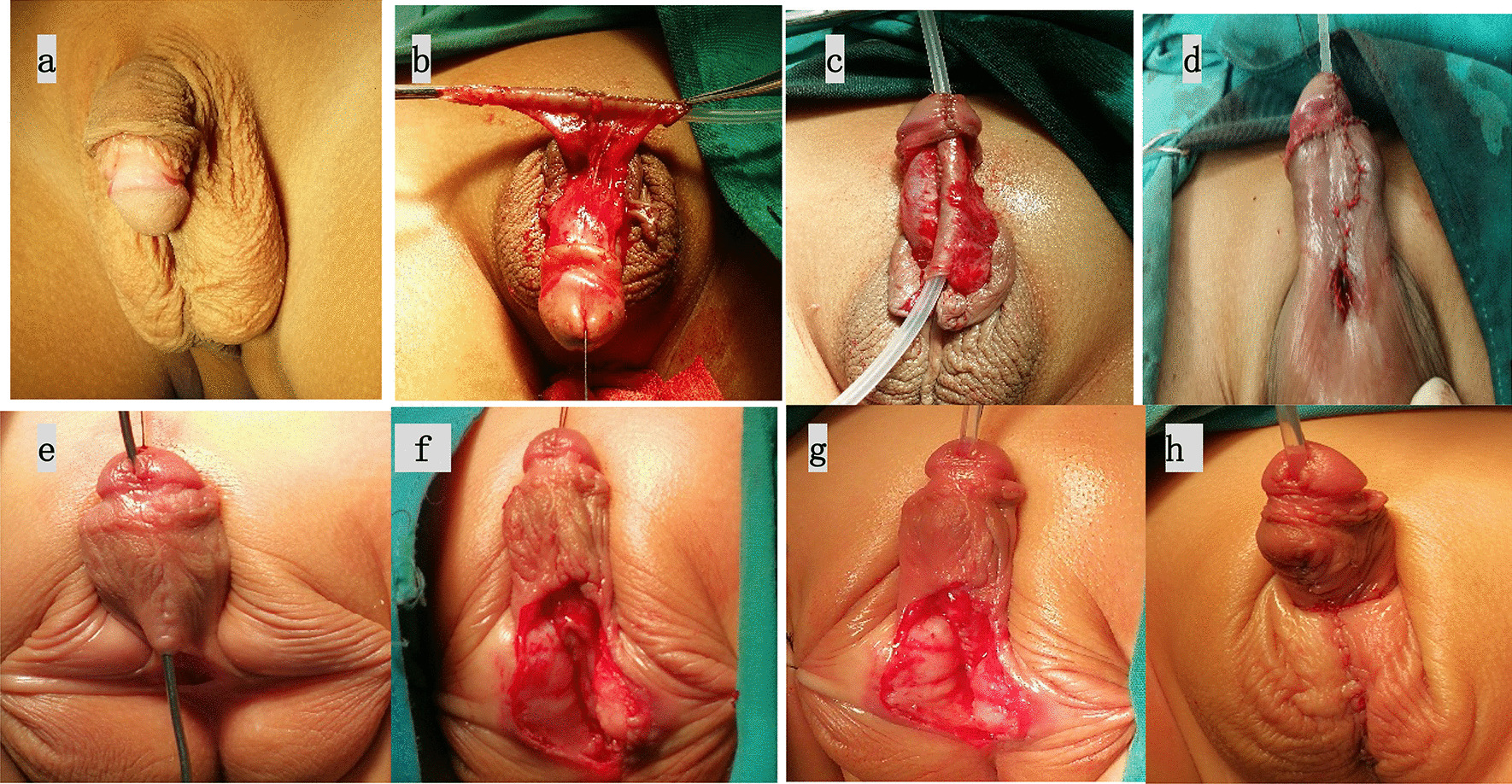
Staging TPIT operation **a** preoperation appearance. **b** Rectangular flap was harvested from the inner prepuce and the mobilized foreskin was rolled into a tube over a catheter; **c** The glans channel was created; **d** The proximal end of the new urethra was anastomosed with the posterior wall of the external urethra, using the anterior wall to form fistula; **e** Preoperation appearance of the second stage; **f**, **g** Urethral fistula was repaired in the second stage; **h** The neourethra was covered by flap

- *Byars staging operation* The first stage is Byars operation. In the first stage, completely straightening ventral curvature was performed firstly. Glanular wings were created by inserting the scissor parallel to the shaft of penis under the glans in midline. The meatus was spatulated. The dorsal preputial skin was then incised in the middle and the two flaps were brought ventrally. Preputial skin flap (Byar’s flap) sutured to cover the raw area created on retraction of the “glans wings” and on areas created after release of chordee. These were sutured to the glans and to each other in the midline of the shaft. The second stage was performed 6–12 months later. Parallel lines were marked on the ventral side of the penis according to the catheter circumference and urethra defect. Incisions were made along these lines to the tip of the glans. The rotated skin was tubularized around the catheter to make the new urethra. The procedure can be seen in Fig. 3.
- *Staging TPIT operation* In the first stage, the transverse island flap was coiled around the catheter to construct the new urethra, the proximal end of the new urethra was anastomosed with the posterior wall of the external urethra, using the anterior wall to form fistula. Urethral fistula was repaired in the second stage. The procedure can be seen in Fig. 4.

### Statistical methods

The data of this study were processed by SPSS22.0 software. A single-factor ANOVA was conducted to analyze the relationship between the age of first operation and urethral defect length; Chi-square test were used for other factors to assess the differences between the groups. The statistical values for the above analyses were significant at P < 0.05. A binomial logistic regression was chosen to determine the relationship between each complication and possible associated risk factors as the independent variables with a defined confidence interval of 95% and significant value of p < 0.05.

## Results

### The DSD classification of the 110 cases reviewed in this study (Table 1)

**Table 1:** DSD classification

DSD classification		N	Sum
46XY, DSD	Complete or partial gonadal dysgenesis	13	58
	Ovotesticular DSD	1	
	5-α reductase deficiency	12	
	Complete and partial androgen insensitivity	1	
	No definitive diagnosis	31	
46XX, DSD	Ovotesticular DSD	17	19
	No definitive diagnosis	2	
Sex chromosomal DSD	45X	1	33
	47, XXY	5	
	45, X/46, XY	27	

Among the 110 cases, there were 58 cases of 46XY DSD including 13 gonadal dysgenesis DSD, 12 androgen synthesis disorder, 1 androgen insensitivity syndrome and 31 unknown etiology. There were 19 cases of 46XXDSD, of which 17 were Ovotesticular oocyte type DSD and 2 were unknown etiology. The rest consisted of 1 case of 45XO DSD, 5 cases of 47XXY DSD, 27 cases of 45XO/46XY DSD.

### The clinical details of the 110 cases reviewed in this study (Table 2)

**Table 2:** Clinical details of the 110 cases

		Urethra percutaneous fistula		Urethral diverticulum		Urethral stricture		Successful operation	
		N	P	N	P	N	P	N	P
Age	37.28 ± 30.67 months (13–184 m)		0.006**		0.451		0.535		0.154
Length of urethral defect	7.74 ± 1.43 cm (0.5–9 cm)		0.001**		0.350		0.242		0.026*
Hypospadias index	Mild to moderate	4	0.444	2	1.000	0	0.692	7	0.689
	Severe	17		12		7		62	
Cryptorchidism	Yes	17	0.067	8	0.589	3	0.438	43	0.709
	No	4		6		4		26	
Small penis	Yes	12	0.863	6	0.310	4	1.000	39	0.770
	No	9		8		3		30	
Gonad	Testis	9	0.239	9	0.121	4	0.861	36	0.911
	Dysplastic gonad	4		4		2		19	
	Ovotestis	7		1		1		11	
	Others	1		1		0		3	
Chromosome	46XY	10	0.828	8	0.398	3	0.806	36	0.994
	46XX	5		1		2		13	
	Others	6		5		2		20	
Preoperative hormone therapy	Yes	7	0.059	8	0.670	4	1.000	38	0.376
	No	14		6		3		31	
Penile scrotum transposition	Yes	6	0.585	3	0.464	2	1.000	26	0.244
	No	15		11		5		43	
Staged surgery	Primary	9	0.915	11	0.003**	4	0.650	23	0.019*
	Staged	12		3		3		46	
TPIT-related surgery	Yes	11	0.000***	10	0.120	7	0.254	64	0.000***
	No	10		4		0		3	
Urethral covering material	No	8	0.097	4	0.620	1	0.574	13	0.318
	Subcutaneous fascia	12		9		4		44	
	Scrotal sarcoid	1		1		2		12	
Catheter removal time	≤ 2 weeks	1	1.000	0	0.851	0	1.000	4	0.731
	≥ 3 weeks	20		14		7		65	

Bold indicates stastical significance

*P < 0.05; **P < 0.01; ***P < 0.001

The success rate of operation was related to the length of urethral defect after a thorough correction of chordee (P = 0.026 < 0.05) and surgical strategy (staged surgery P = 0.019 < 0.05, TPIT-related surgery P = 0.000 < 0.05). The occurrence of urethral fistula was related to the age of the first operation (P = 0.006 < 0.05), the length of urethral defect after the thorough correction of chordee (P = 0.001 < 0.05) as well as the surgical strategy (TPIT related P = 0.000 < 0.05). The incidence of postoperative urethral diverticulum was only related to surgical strategy (Staged surgery P = 0.003 < 0.05).

### The success rate of operation and the basic situation of postoperative complications (Fig. 5)

**Fig. 5:**
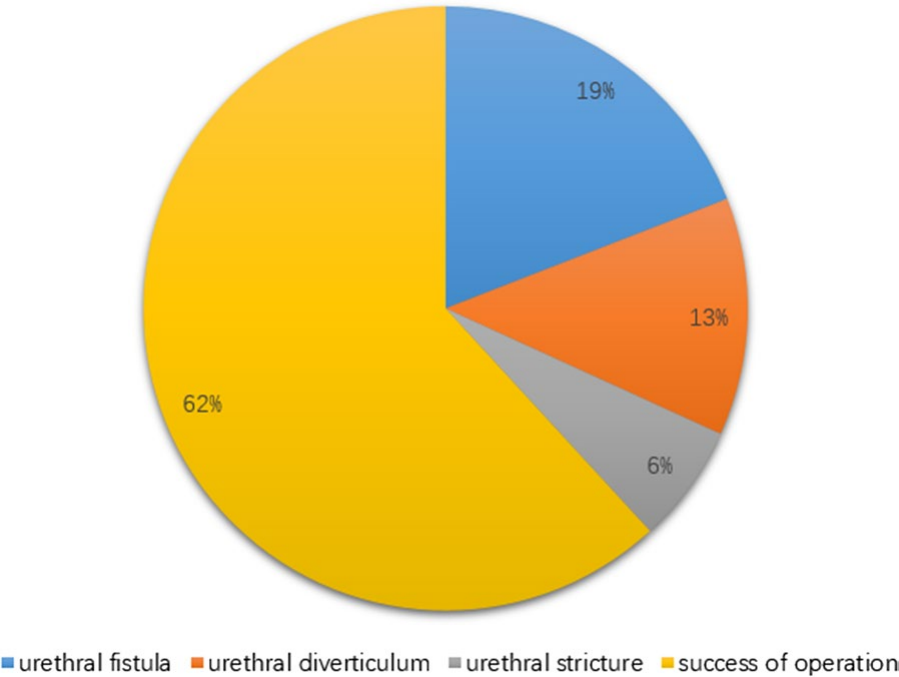
The success rate of operation and the basic situation of postoperative complications. Among the 110 cases of DSD, 62% belonged to successful operation, 38% had complications in which 19% urethrocutaneous fistula, 13% urethral diverticulum, and 6% urethral stricture

Among the 110 cases of DSD, 62% belonged to successful operation, 38% had complications in which 19% urethrocutaneous fistula, 13% urethral diverticulum, and 6% urethral stricture. Other common complications, such as the chordee and dehiscence), were not observed during the study.

### The correlation analysis between the main influencing factors and the success rate of operation and postoperative complications results

**Fig. 6:**
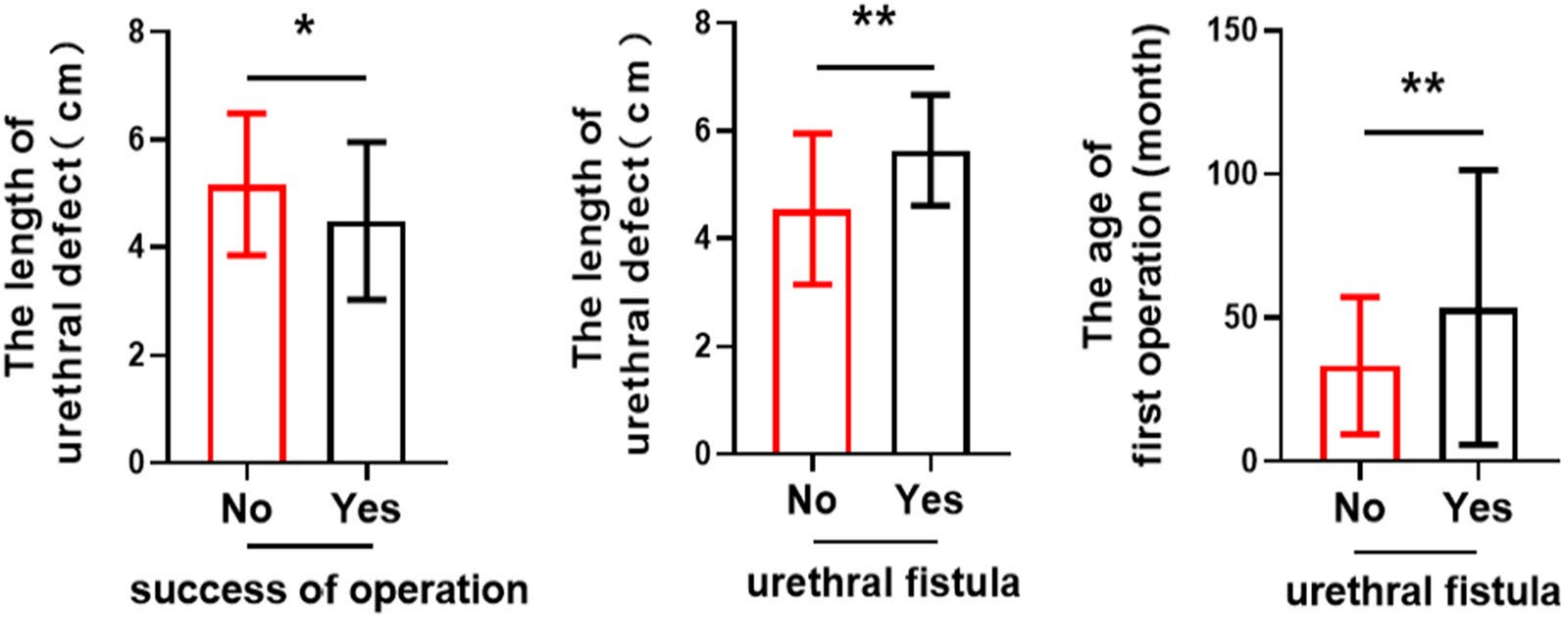
The correlation analysis between the risk factors and the effect of operation. The success rate of operation was relative to the length of urethral defect (P = 0.005 < 0.05). Urethrocutaneous fistula was related to the age of first operation (P = 0.006 < 0.05), the length of urethral defect (P = 0.005 < 0.05)

**Fig. 7:**
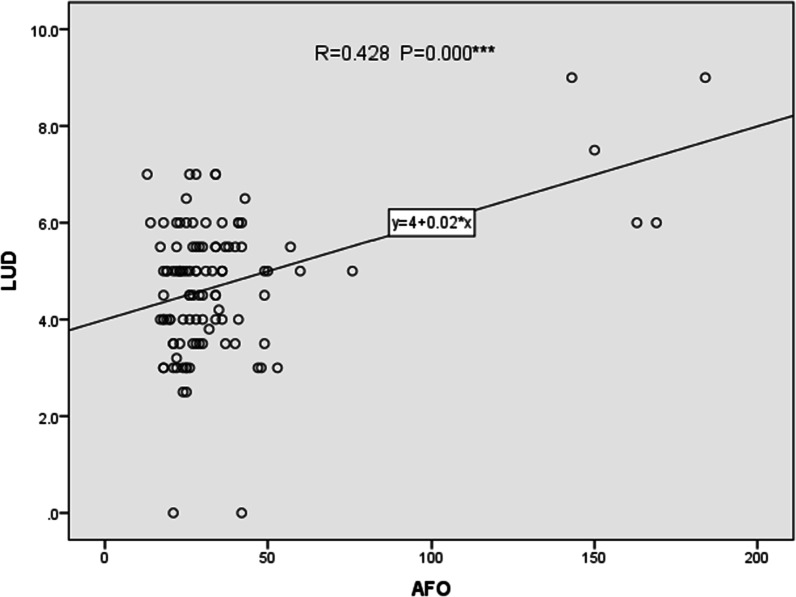
Relationship of the age of first operation (AFO) and the length of urethral fistula. The age of first operation (AFO) and the length of urethral fistula are related (P = 0.000 < 0.05)

1. The success rate of operation was relative to the length of urethral defect (P = 0.005 < 0.05), surgical strategies (staged operation P = 0.019 < 0.05, TPIT-related operation P = 0.000 < 0.05). Urethrocutaneous fistula was related to the age of first operation (P = 0.006 < 0.05), the length of urethral defect (P = 0.005 < 0.05), and TPIT-related operation (P = 0.000 < 0.05). Urethral diverticulum was only related to staged operation (P = 0.003 < 0.05). Details are shown in Table 2 and Fig. 6.
2. The correlation analysis between other factors and the success rate of operation as well as postoperative complications were not statistically significant (P > 0.05). There is no significant relationship between urethral stricture and any of the factors (P > 0.05).
3. The relationship of the age of first operation and the length of urethral defect is shown in Fig. 7.

The result showed that the age of first operation and the length of urethral defect were related.

### Logistic regression analysis

The results of logistic regression analysis of the factors related to the success rate of operation, urethral fistula, urethral diverticulum were shown in Table 3.

**Table 3:** Logistic regression analysis of the factors related to the success rate of operation, urethral fistula, urethral diverticulum

	Length of urethral defect	TPIT-related surgery	Staged surgery	Surgery age
*Success of operation*				
B	− 0.473	2.293	1.238	–
OR(95% CI)	1.605 (1.157–2.228)	9.905 (2.615–37.511)	0.290(0.120–0.703)	–
P	0.005**	0.001***	0.006**	–
*Urethral diverticulum*				
B	–	–	-1.737	–
OR(95% CI)	–	–	0.173 (0.043–0.714)	–
P	–	–	0.015*	–
*Urethral fistula*				
B	0.579	− 2.507	–	0.012
OR(95% CI)	1.784 (1.090–2.918)	12.273 (3.726–40.425)	–	1.012 (0.995–1.030)
P	0.021*	0.000***	–	0.161
*Urethral stricture*				
B	–	–	–	–
OR(95% CI)	–	–	–	–
P	–	–	–	–

*P < 0.05; **P < 0.01; ***P < 0.001

## Discussion

At present, people pay more and more attention to the treatment of DSD patients. In the past, the urethroplasty among patients with DSD and severe hypospadias was perceived the same way since the treatment was mainly for the repair of hypospadias in DSD patients. In recent years, people gradually realized that DSD patients who underwent urethroplasty were a unique subgroup of hypospadias patients that needed to be studied and analyzed separately [6]. Previous studies on proximal hypospadias had shown that the factors affecting the success rate of proximal hypospadias are operative age, suture selection, bleeding control, urine drainage, postoperative infection and so on. This study mainly focused on the main complications of urethroplasty in patients with DSD, such as urethral fistula, urethral stricture, and urethral diverticulum. On the basis of the influencing factors of severe hypospadias, the possible influencing factors were expanded and analyzed based on the characteristics of DSD.

The study found that the factors related to the success rate of operation were the length of urethral defect (P = 0.026 < 0.05) and the method of operation (P = 0.019 < 0.05).

According to the results, we conducted a separate analysis of the factors influencing the three most common surgical complications that require reoperation:

### Urethral diverticulum

The formation of urethral diverticulum could be the result of many factors. After studying urethral diverticulum and various factors, it concluded that the staged operation was related (P = 0.003 < 0.05) but had nothing to do with the length of urethral defect.

The results of this study showed that the staged surgery could decreased the occurrence of urethral diverticulum. The flatness of the foreskin and the stability of the rebuilt urethra during the one-stage operation were worse than the staged surgery since the formed part of the urethra was more stable with urethral plate prepared in the first stage. In the staged surgery, the surgeons directly formed the residual defect of the urethra with the Duplay operation during second-stage surgery, which makes the newly-built urethra more stable than the one-stage operation. It reduced the possibility of urethral diverticulum formation due to the less vortex flow in the urine because of more stable urethra [7].

It wasn’t found that there was obvious correlation between the length of urethral defect and the generation of urethral diverticulum. Liu Xin and her team found that the risk of urethral diverticulum would increase by 2.54 times if the length of the primary urethroplasty increased by one centimetre [8]. It also suggested that patients with defective urethral length above 3.35 cm were more likely to develop urethral diverticulum. However, the correlation was not found in this study.

### Urethrocutaneous fistula

Previous reports on factors affecting urethrocutaneous fistula often included the type of hypospadia, the age of operation, the width of the urethral plate, and various elements of the urethroplasty [9]. Building on the existing data, we did partial verification and found that the occurrence of urethrocutaneous fistula was related to the age of the first operation (P = 0.006 < 0.05), the length of urethral defect (P = 0.001 < 0.05), and TPIT-related operation (P = 0.000 < 0.05). The evidence suggests no correlation with the type of hypospadia, the time of urethral stent, and the coverage with a protective layer.

The data showed the incidence rate of the urethrocutaneous fistula elevated as the urethra defect increased. It was analyzed that the longer the defective urethra was, the more urethral repair materials were needed, and the greater the aspect ratio of the skin flap was used to repair the urethra. Thus, a longer urethral defect made it difficult to ensure a sufficient blood supply, bringing hardship to the growth of the flap and the healing of the anastomosis. What’s more, a longer reconstructed urethra produced more anastomoses during the operation, resulting in more complications such as urethrocutaneous fistula [10]. Regarding the age of first operation, additional analysis was carried on the relationship between the age of first operation and the length of urethral defect. The two had a significant correlation (P = 0.006 < 0.05) while the P-value of logistic regression between age and urethrocutaneous fistula was 0.161 > 0.05. Although the age of the first operation was related to the occurrence of postoperative urethral fistula, it was not an independent factor. In the studies [11–13] on the related factors of urethrocutaneous fistula after hypospadias, the influence of the age of the first operation on the effect of operation was differed. Some people thought that the older the age of operation, the greater the possibility of postoperative complications, especially for urethrocutaneous fistula [14]. However, several experts thought that operation age on the effect of operation was not significant within a certain age range. It suggested the success rate of operation decreased with the increase of age before a certain age. Therefore, it was possible that the influence of operation age existed, but with certain limitation.

In addition, this study also found that TPIT-related surgery was beneficial for the occurrence of urethrocutaneous fistula. TPIT was not accepted by surgeons because of its complex operation and long learning curve. TPIT required higher blood supply of skin flap because of its circular anastomosis and island flap, so the possibility of urethrocutaneous fistula after operation should be higher. However, the actual result was different from the assumption. The primary surgery was mainly TPIT and Koyanagi. There was one longitudinal anastomosis line attached to the cavernosum of the penis and the coverage of the urethral anastomosis was paid attention to in the TPIT procedure. However, there were two longitudinal anastomosis lines, one of which was not covered by anything in the Koyanagi operation. Because of that, the occurrence rate of postoperative urethrocutaneous fistula in Koyanagi was higher. In the staged procedure, the second stage of Byars staged surgery was urethroplasty while the second stage of staging TPIT surgery was the repair of urethrocutaneous fistula. The length of the urethral anastomosis in staged TPIT surgery was much shorter than Byars staged surgery, lowering the possibility of postoperative urethrocutaneous.

It must be mentioned that the study of the relationship between the surgical success rate and staged surgery suggested that there was a significant correlation between the two. However, in the correlation study between urethrocutaneous fistula and staged surgery, it was found that there was no significant correlation. Although staged surgery increased the success rate of surgery, it didn’t reduce the incidence of postoperative urethrocutaneous fistula. In theory, staged surgery ensured the blood supply of the newly-built urethra which leading to further reduce the occurrence of urethrocutaneous fistula, but an analysis of the two showed no correlation—staged surgery didn’t reduce the occurrence of urethrocutaneous fistula. It was believed that the influence wasn’t obvious that staged surgery brought in the blood supply of the newly-built urethra. The major problem resulting in urethrocutaneous fistula was the blood supply of anastomoses.

It wasn’t found that the urethrocutaneous fistula was related to the covering layer of the reconstructed urethra, different from several previous article [9, 15]. It suggested a lower possibility of urethrocutaneous fistula occurred if the testicular sheath covered the urethra, following by scrotal sarcoid coverage and subcutaneous fascia coverage in these articles. In this study, the correlation P-value between the two is 0.061, closer to P < 0.05. According to the rate of postoperative urethrocutaneous fistula, the possibility of urethrocutaneous fistula with the urethra covered by the scrotal sarcoid was less than that covered by the subcutaneous fascia, both better than without any coverage. Different results may be obtained if the sample size was further expanded.

The correlation analysis of the occurrence of urethrocutaneous fistula and the use of hormones came to a result of P = 0.059 (relatively close to P < 0.05). Previous reports had also suggested that the use of hormones can promote blood supply to the skin flaps, hence reducing the occurrence of postoperative urethrocutaneous fistula [16]. The sample size could be further expanded to verify whether the two are related.

### Urethral stricture

In the study, it came to the conclusion that there was no relationship between urethral stricture and all factors.

Urethral stricture was one of the common complications after urethroplasty, which usually occurred within 6 months after operation, even for a longer time such as in sexual development or adulthood [17]. The follow-up time of all cases in this study was more than 1 year. However, all cases had not been followed up until sexual development, so the study lacks a certain degree of rigor. As for the influence of surgical methods on urethral stricture, experts believed that urethral stricture after urethroplasty often occurs in urethroplasty with pedicled skin flap or free tissue coiled tube, because there was a circular anastomosis in this kind of operation [6, 7]. Circular scar contracture was easy to occur at the anastomotic site, leading to urethral stricture. However, no correlation between surgical methods and urethral stricture was found in this study. It had to be noted that all urethral strictures in this study occurred in TPIT-related procedures, and there were 91 cases of TPIT-related operations in this study, accounting for 82.7% of the total cases. Therefore, the deviation in the number of cases may affect the statistical results. In future studies, we needed to further balance the number of cases between the two groups, and further statistical analysis might lead to different results.

## Conclusion

This study supports that the influencing factors for urethroplasty on DSD patients mainly include the length of the urethral defect, the operation method. The length of the urethral defect mainly affects the occurrence of postoperative urethrocutaneous fistula. The longer the urethral defect, the more likely postoperative urethrocutaneous fistula will occur. The surgical method affects the occurrence of both postoperative urethrocutaneous fistula and postoperative urethral diverticulum. Staged surgery can reduce the occurrence of postoperative urethral diverticulum, but has no obvious impact on the production of postoperative urethrocutaneous fistula. Urethrocutaneous fistula is less in TPIT-related surgery.

This paper is only a preliminary analysis of the factors related to the urethroplasty on DSD patients. A total of 110 cases over four years from 2016 to 2019 were collected. The number of cases is limited. Other related factors listed in this article would be different if we expand the sample size. Moreover, this article is based on complications within one to four years. The evaluation time is short, and many delayed complications may not have appeared yet.

In addition, this article focuses on analysing complications such as urethral fistula, urethral stricture, and urethral diverticulum, which are very critical for a successful treatment. Whether there is a recurrence of penile curvature, satisfaction with the appearance of the penis, urination, sexual intercourse, and other factors has not yet been studied, and further long-term follow-up is needed [18].

## Data Availability

The original data during the current study were unavailable for other institute. We have signed an agreement with parents not to disclose the basic medical information of patients to anyone outside our hospital when applying for ethics.
